# Hemophagocytic Lymphohistiocytosis (HLH) or Macrophage Activation Syndrome (MAS)? A Lethal Case of Malignancy-Associated Hemophagocytic Lymphohistiocytosis in a Patient With Concurrent Autoimmune Disease

**DOI:** 10.7759/cureus.98078

**Published:** 2025-11-29

**Authors:** Michael Cargill, Cristine K Arcilla, Gurjit S Kaeley, Myint Thway, Ahmad Alkhasawneh, Raafat Makary, Ifra Badar

**Affiliations:** 1 Department of Medicine, University of Florida College of Medicine – Jacksonville, Jacksonville, USA; 2 Department of Rheumatology, University of Florida College of Medicine – Jacksonville, Jacksonville, USA; 3 Department of Pathology, University of Florida College of Medicine – Jacksonville, Jacksonville, USA

**Keywords:** adult t cell lymphoma, diagnosis difficulty, hemophagocytic lymphohistiocytosis (hlh), hyperinflammatory syndrome, macrophage activation syndrome (mas)

## Abstract

Hemophagocytic lymphohistiocytosis (HLH) is a life-threatening hyperinflammatory syndrome caused by uncontrolled macrophage and T-cell activation leading to multiorgan failure. While primary HLH has a genetic basis, secondary HLH can arise from various triggers. Macrophage activation syndrome (MAS) is a subtype of HLH associated with autoimmune disorders, whereas malignancy-associated HLH (mHLH) is typically linked to cancers, particularly T-cell lymphomas. HLH is frequently underrecognized and may initially be mistaken for MAS due to overlapping clinical and laboratory findings, especially in the context of an occult malignancy. We present a 58-year-old male who presented with fever, cytopenias, and transaminitis and met six of the HLH-2004 diagnostic criteria. MAS was the initial working diagnosis; however, a bone marrow biopsy demonstrated hemophagocytosis and T-cell lymphoma, confirming mHLH. Despite targeted treatment, the patient experienced progressive multiorgan failure and was transitioned to hospice care. This case highlights the diagnostic complexity of HLH and the importance of distinguishing mHLH from MAS. Because therapeutic strategies differ based on the predominant trigger, autoimmune versus malignant, early recognition and accurate classification are essential to optimize outcomes and avoid delays in appropriate therapy.

## Introduction

Hemophagocytic lymphohistiocytosis (HLH) is a rare, life-threatening hyperinflammatory syndrome caused by dysregulated activation of macrophages, CD8⁺ T cells, and natural killer (NK) cells, leading to an excessive immune response that can result in multiorgan failure and death. HLH typically presents as a febrile illness with multiorgan involvement, including splenomegaly, transaminitis, cytopenias, and decreased NK cell activity. The diagnosis of HLH is established by fulfilling at least five of the eight HLH-2004 diagnostic criteria: fever, splenomegaly, bicytopenia, hypertriglyceridemia and/or hypofibrinogenemia, hemophagocytosis, hyperferritinemia (>500 mg/L), elevated soluble CD25 (>2400 U/mL), and decreased NK cell activity [1].

Primary HLH typically presents in early childhood and is attributed to genetic mutations that impair cytotoxic interactions between NK cells, CD8⁺ T cells, and antigen-presenting cells (APCs), resulting in uncontrolled immune activation. Secondary HLH shares a similar pathophysiology but arises from external triggers rather than inherited defects. Macrophage Activation Syndrome (MAS) represents a subset of HLH driven by autoimmune disorders, most commonly adult-onset Still’s disease [2]. Whereas malignancy-associated HLH (mHLH) is primarily linked to cancers, particularly T-cell lymphomas [3]. Other triggers of secondary HLH include infections (e.g., tuberculosis, Epstein-Barr virus, and fungal infections), certain monoclonal antibody therapies, and transplantation [4].

Early recognition of HLH is critical, as treatment strategies differ depending on the underlying etiology. For malignancy-associated or refractory HLH, therapy typically follows the HLH-94 protocol, which includes etoposide and dexamethasone followed by cyclosporine A [1,5]. Intravenous immunoglobulin may be considered in milder cases, and treatment of the underlying malignancy is often required, with etoposide sometimes incorporated into CHOP-like regimens. This differs from MAS management, where high-dose glucocorticoids and IL-1 antagonists are first-line therapies targeting the autoimmune trigger [6]. Screening for malignancy prior to prolonged MAS-directed therapy is essential, given the potential suppression of immune surveillance. In steroid- or IL-1-resistant MAS, reduced-dose etoposide may also be beneficial.

We report the case of a 58-year-old male with a history of recurrent autoimmune hemolytic anemia who presented with clinical and laboratory findings suggestive of MAS. Subsequent evaluation revealed underlying T-cell lymphoma, confirming mHLH, and malignancy-directed therapy was initiated. Despite treatment, the patient’s condition deteriorated, leading to multiorgan failure and transition to hospice care. Given the distinct therapeutic strategies for HLH and MAS, this case underscores the lethality of HLH and the importance of early recognition, accurate differentiation, and prompt treatment.

## Case presentation

A 58-year-old male with a past medical history of pulmonary nodules, mediastinal lymphadenopathy, and recurrent autoimmune hemolytic anemia presented to the emergency room with generalized weakness. He reported recurrent episodes of pneumonia over the past four months and a 10-lb weight loss in the last month. Additional symptoms included progressive weakness, exertional dyspnea, and increasing fatigue with minimal exertion.

On admission, he was afebrile, tachycardic (121 bpm), and with otherwise normal vital signs. Labwork was notable for leukopenia, anemia, thrombocytopenia, hyponatremia, and transaminitis (Table 1).

**Table 1:** Patient laboratory values. WBC: White Blood Cells, AST: Aspartate Aminotransferase, ALT: Alanine Aminotransferase, ESR: Erythrocyte Sedimentation Rate, CRP: C-Reactive Protein Table 1: Patient’s laboratory profile demonstrating cytopenia, hyperferritinemia, hypertriglyceridemia, and elevated IL-2 receptor antibody consistent with HLH criteria

Parameter	Patient Laboratory Values	Reference Values
WBC	3.11 thou/ccm	4.5-11 thou/ccm
Hemoglobin	10.6 g/dL	14.0-18.0 g/dL
Platelets	133,000 per µL	140,000-440,000 per µL
AST	46 U/L	14-33 U/L
ALT	59 U/L	10-42 U/L
Alkaline Phosphatase	245 U/L	40-129 U/L
Ferritin	15,240 ng/ml	30.0-400.0 ng/ml
Triglycerides	238 mg/dl	<150 mg/dl
ESR	16 mm/hr	0-15 mm/hr
CRP	75.70 mg/L	0.00-5.00 mg/L
Fibrinogen	162 mg/dL	186-461 mg/dL
Soluble Interleukin-2 receptor antibody	11973 U/ml	223-710 U/ml
Absolute NK cells	72 cells/µL	24-406 cells/µL

**Figure 1:**
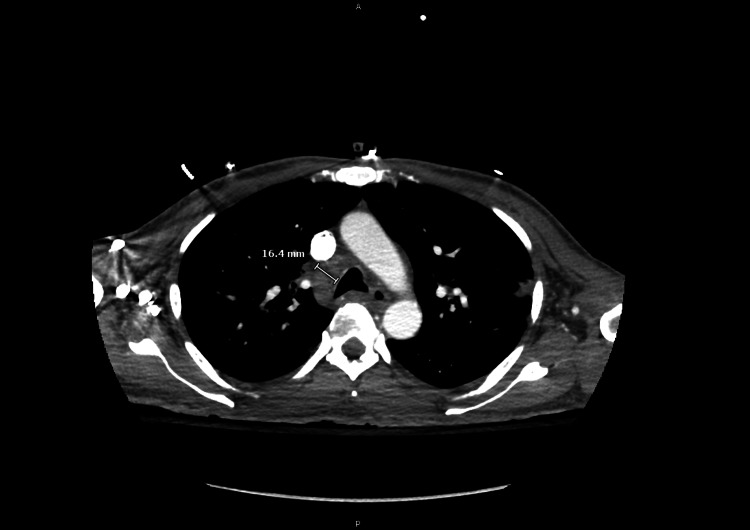
CTA of the chest demonstrating mediastinal and hilar lymphadenopathy. Image 1: CTA of the chest demonstrating mediastinal and hilar lymphadenopathy. The patient had been under outpatient pulmonology follow-up for waxing and waning pulmonary nodules. A bronchoscopy with endobronchial ultrasound (EBUS) performed one year prior revealed lymphohistiocytic elements, without evidence of malignancy. These findings were considered suggestive of an inflammatory process, with differential considerations including organizing pneumonia, hypersensitivity pneumonitis, chronic eosinophilic pneumonia vs. other autoimmune etiologies.

A chest computed tomography angiography (CTA) revealed multiple known pulmonary nodules that had increased in size compared to several months earlier (Figure 1).

Of note, his pulmonary nodules and mediastinal lymphadenopathy had been evaluated one year prior with endobronchial ultrasound (EBUS), which was negative for malignancy but demonstrated lymphohistiocytic elements, although it was non-diagnostic for granuloma. These findings were suggestive of an inflammatory process, and the patient had follow-up appointments scheduled to further evaluate the nodules prior to his admission. During this admission, the prior workup contributed to a greater suspicion for an autoimmune etiology/MAS and initially lowered concern for malignancy, illustrating the diagnostic challenge central to this case.

Before receiving empiric antibiotics, the patient became tachycardic and hypotensive, refractory to intravenous fluids, and required vasopressors for several days. He also developed persistent fevers despite a negative infectious workup. There was a strong suspicion of a hyperinflammatory syndrome given his elevated ferritin level of 15,240 µg/L, fevers, thrombocytopenia, and hypertriglyceridemia (238 mg/dL). Considering his history of autoimmune hemolytic anemia, negative malignancy evaluation, and prior inflammatory findings related to his pulmonary nodules, an autoimmune process was suspected, and a working diagnosis of Macrophage Activation Syndrome (MAS) was made.

**Figure 2:**
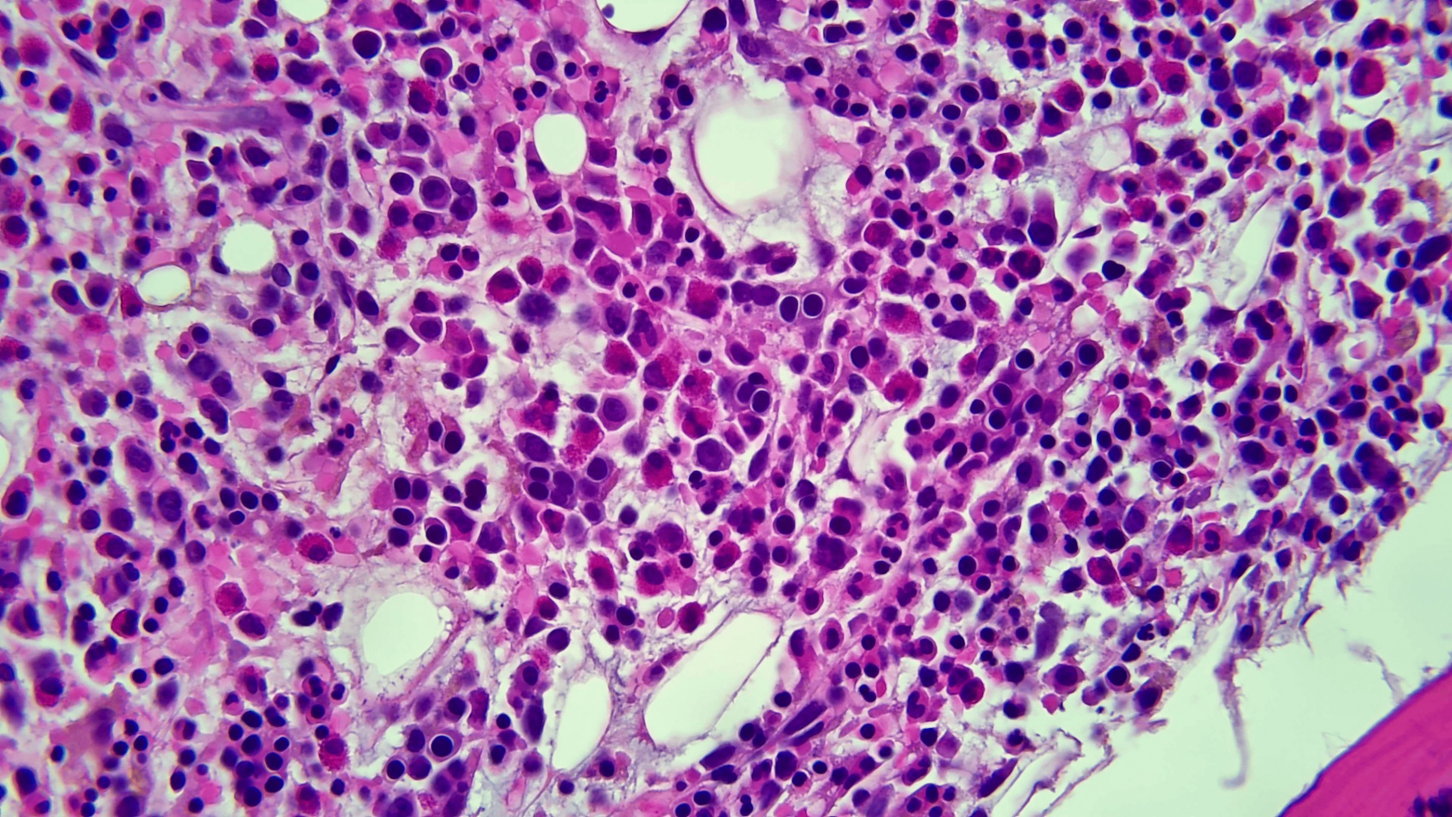
Bone marrow biopsy demonstrating atypical T-cells with increased eosinophils.

**Figure 3:**
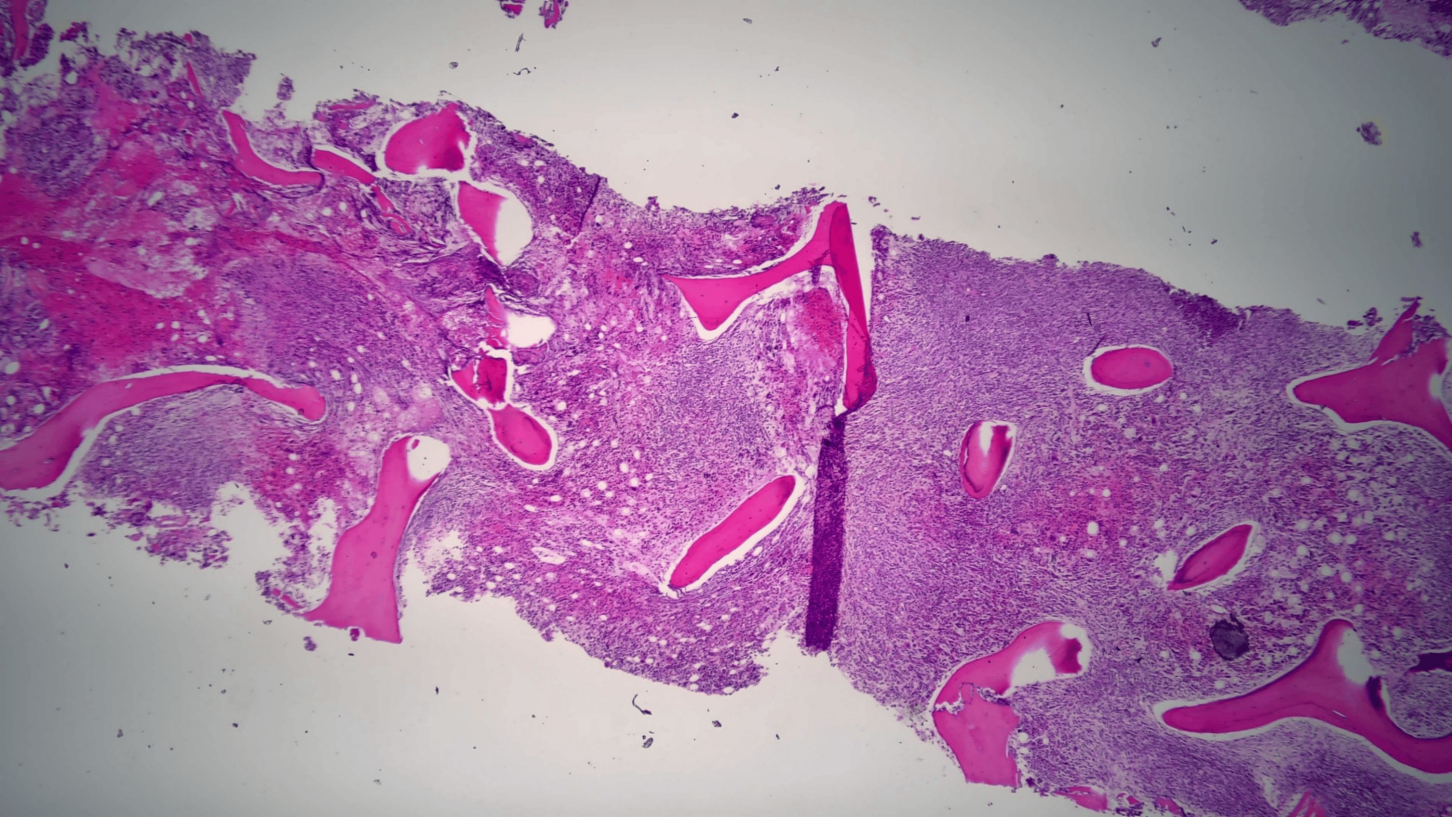
Bone marrow biopsy demonstrating hypercellular marrow.

**Figure 4:**
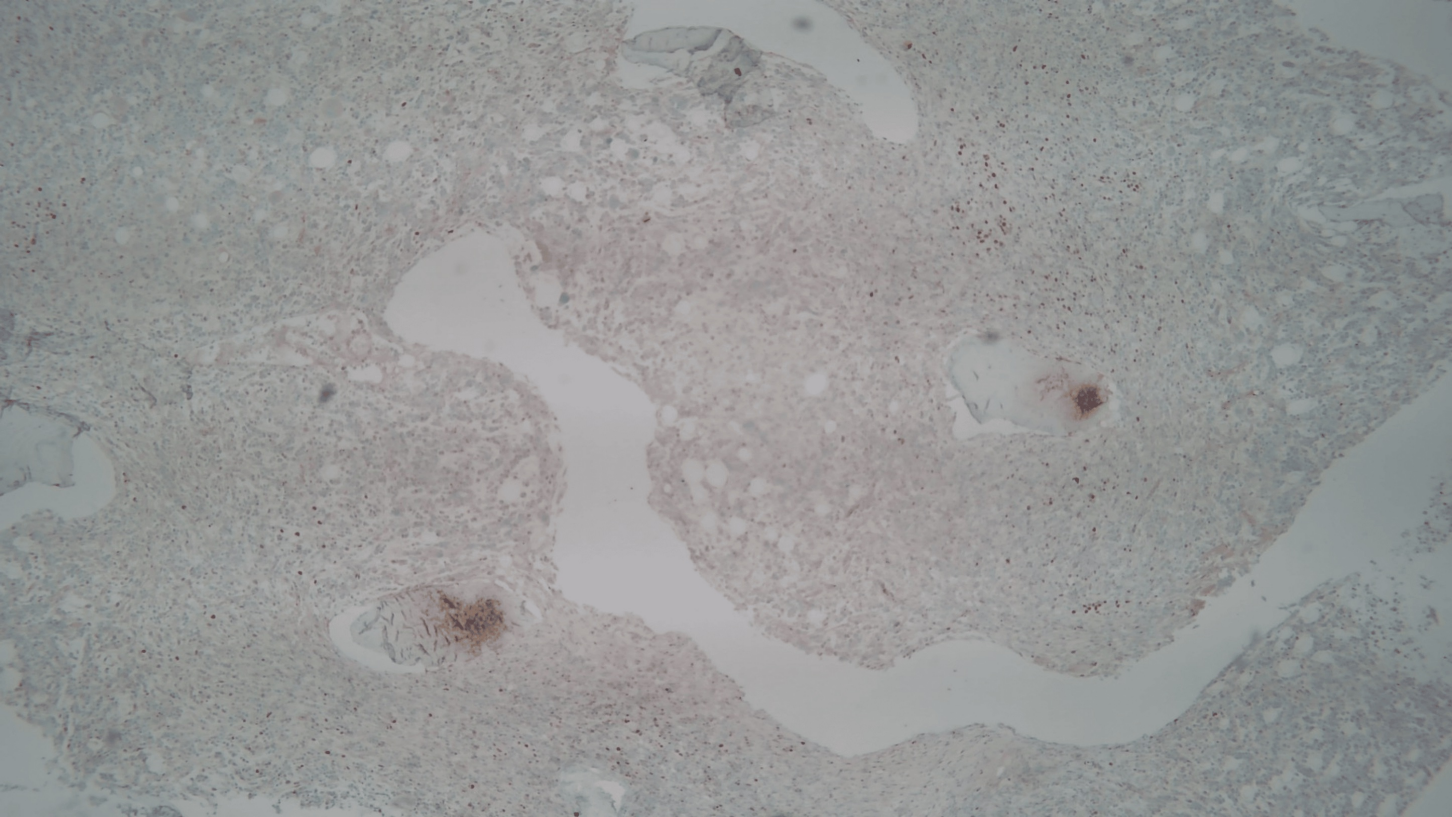
Bone marrow biopsy demonstrating a subset of T-cells with BCL6 positivity.

Further laboratory tests revealed low ESR, low fibrinogen, elevated CRP, elevated soluble interleukin-2 receptor alpha, and elevated CD25, with negative antinuclear antibody (ANA), antineutrophil cytoplasmic antibodies (ANCA), and cyclic citrullinated protein (CCP). Finally, a bone marrow biopsy was obtained and subsequently revealed hemophagocytosis and findings consistent with T-cell lymphoma (Figures 2-4).

Plans were made to initiate steroid-interleukin-1 therapy; however, his history of incarceration and an indeterminate QuantiFERON test on admission raised concern for latent tuberculosis. A repeat QuantiFERON test also returned indeterminate, prompting bronchoscopy with AFB testing and fungal blood cultures prior to initiating immunomodulatory therapy. The patient was transferred to a tertiary medical center nine days after the initial HLH concern was raised in preparation for treatment. Following confirmation of malignancy on bone marrow biopsy, he was diagnosed with malignancy-associated hemophagocytic lymphohistiocytosis and began mHLH-directed therapy with dexamethasone and etoposide on the tenth day of admission, with plans for subsequent lymphoma-directed treatment following HLH suppression. Despite these efforts, his clinical condition worsened, with waxing and waning mental status, intermittent fevers, tachycardia, and worsening cytopenias that required multiple blood transfusions. His respiratory and hemodynamic status continued to deteriorate despite two cycles of etoposide and daily high-dose steroids. After a discussion with the patient and his family, he was transitioned to hospice care and passed away the day after discharge.

## Discussion

Hemophagocytic lymphohistiocytosis (HLH) and macrophage activation syndrome (MAS) are rare hyperinflammatory conditions caused by dysregulated activation of macrophages, CD8+ T cells, and NK cells, leading to multiorgan failure and death. HLH is classified as primary (genetic) or secondary (acquired). Primary HLH typically presents in early childhood and is attributed to inherited genetic conditions related to impaired interactions between NK cells, CD8+ T cells, and antigen-presenting cells (APCs). Secondary HLH generally occurs in adults and is triggered by an acute illness, such as infection, malignancy, or autoimmune disorders. MAS is a specific subgroup of secondary HLH in which an underlying autoimmune disease is the predominant trigger. The diagnosis of an HLH subtype depends on the predominant trigger when two possible triggers are present. In this case, the patient’s history of a recurrent autoimmune condition, prior negative malignancy evaluation for his pulmonary nodules, and previously noted inflammatory findings initially supported a diagnosis of MAS, given the higher suspicion for an autoimmune etiology. However, after the subsequent discovery of his T-cell lymphoma, the diagnosis was revised to mHLH, and the patient was started on mHLH-directed therapy.

Estimating the incidence of mHLH and MAS remains challenging due to frequent underdiagnosis. However, one tertiary medical center estimated the occurrence of HLH at approximately 1 in 2000 adult critical care admissions, with 52% of cases classified as mHLH, 32% related to infection, and 8% associated with an underlying autoimmune disorder [7]. In another study, mHLH was reported to have an annual incidence of fewer than 0.4 cases per 100,000 adults in Sweden [3]. A review of lymphoma-associated HLH found that T-cell non-Hodgkin lymphomas were present in 45.2% of cases [8]. Such a close relationship may be due to a dysregulated immune response and cytokine storm induced by malignant T-cells. Regarding MAS, reported cases in adults are similarly limited. A retrospective literature review from 2007 to 2017 identified seven adult cases of MAS complicating rheumatologic diseases, with adult-onset Still’s disease and systemic lupus erythematosus being the most common autoimmune conditions associated with MAS [2]. In five MAS patients over a 16-year period at one tertiary medical center, three presented with systemic lupus erythematosus, one with a mixed connective tissue disorder, and one with Kikuchi disease [7]. Clinicians may use this information to better stratify their approach to HLH subgroups, ensuring that underlying malignancies are thoroughly ruled out, and recognizing that among autoimmune conditions associated with MAS, adult-onset Still’s disease and systemic lupus erythematosus are most frequently implicated.

A diagnosis of HLH is made by meeting at least five of the eight HLH-2004 diagnostic criteria, which include fever, splenomegaly, cytopenia affecting >2 of 3 peripheral blood lineages (hemoglobin <90 g/L, platelets <100 × 10^9/L, neutrophils <1.0 × 10^9/L), hypertriglyceridemia (fasting triglycerides >3.0 mmol/L) and/or hypofibrinogenemia (fibrinogen <1.5 g/L), hemophagocytosis (in bone marrow, spleen, or lymph nodes), hyperferritinemia (>500 μg/L), elevated soluble CD25 (>2400 U/mL), and decreased NK cell activity [1]. Additional lab findings may include elevated CRP, low ESR (due to fibrinogen consumption), transaminitis, hyperbilirubinemia, and elevated lactate dehydrogenase. Additionally, a ferritin:ESR ratio of 11:3 has been demonstrated as a potential method of differentiating MAS from systemic infections in systemic juvenile idiopathic arthritis [9]. In this case, the patient met six of the eight HLH-2004 diagnostic criteria. 

Treatment of HLH depends on the underlying etiology. For malignancy-associated or resistant HLH, treatment typically follows the HLH-94 protocol, which includes etoposide and dexamethasone for the initial eight weeks, followed by cyclosporine A after nine weeks [1,5]. Additional therapies may include IV immunoglobulin (in milder cases), emapalumab (anti-IFN-γ antibody), tocilizumab (IL-6 blockade), and treatment of underlying malignancy. Etoposide, a topoisomerase II inhibitor, induces apoptosis of rapidly dividing cells and selectively depletes T cells and macrophages, thereby controlling T-cell activation and cytokine production. Dexamethasone reduces cytokine and lymphocyte production. IV immunoglobulin may also be considered in milder cases, and the treatment of the underlying malignancy may be necessary, with etoposide sometimes added to CHOP-like protocols. Second-line treatment options include the anti-IFN-γ antibody emapalumab, and evidence for IL-6 blockade agents (e.g., tocilizumab) is also increasing [1]. This differs slightly from the treatment of MAS, in which high-dose glucocorticoids and IL-1 antagonists are often first-line therapies for addressing the underlying autoimmune condition. In particular, IL-1 antagonists such as anakinra have demonstrated the ability to induce disease remission and improve survival by targeting cytokine storms through IL-1R blockade on T cells and inhibiting downstream inflammatory signaling pathways [6]. Notably, screening for malignancies before initiation is crucial, given the potential suppression of immune surveillance with prolonged treatment. Finally, in MAS cases refractory to steroids and IL-1 antagonists, a reduced dose of etoposide may be effective. The success of the HLH-94 protocol has been shown to result in a 54% five-year survival rate [10]. Several factors, such as a lack of early response to therapy (e.g., improvement in sCD25, platelet count, absolute lymphocyte count, and BUN by day 7), older age, EBV-associated HLH, thrombocytopenia, and delayed administration of etoposide, are associated with poorer outcomes [11].

## Conclusions

In this case, a 58-year-old male initially presented with clinical and serological findings suggestive of MAS. However, further evaluation revealed underlying T-cell lymphoma, and the patient was subsequently started on directed therapy for mHLH. He had several poor prognostic factors, including thrombocytopenia, delayed etoposide administration due to a prolonged diagnostic consideration of MAS, and a lack of early response to therapy. The patient was transitioned to hospice care before completing the HLH-94 protocol due to progressive clinical deterioration despite treatment. Given the rarity of HLH and the diagnostic uncertainty created by overlapping inflammatory and malignant conditions, this case underscores the critical importance of prompt recognition, early subtype identification, and the use of epidemiologic data to differentiate mHLH from MAS and to initiate timely, targeted treatment for this underdiagnosed and life-threatening condition.
